# Datasets for the microstructure of nanoscale metal network structures and for its evolution during coarsening

**DOI:** 10.1016/j.dib.2019.105030

**Published:** 2019-12-24

**Authors:** Yong Li, Bao-Nam Dinh Ngô, Jürgen Markmann, Jörg Weissmüller

**Affiliations:** a Institute of Materials Physics and Technology, Hamburg University of Technology, Hamburg, Germany; b Institute of Materials Research, Materials Mechanics, Helmholtz-Zentrum Geesthacht, Geesthacht, Germany

**Keywords:** Nanoporous metal, Bicontinuous microstructure, Network structure, Coarsening, Topological genus, Spinodal decomposition, Surface energy anisotropy, Kinetic Monte Carlo simulation

## Abstract

The datasets in this work are files containing atom position coordinates of volume elements approximating nanoporous gold made by dealloying and annealing. The material is represented in an as-prepared state and in various stages of coarsening, as described in Phys. Rev. Mater, 3 (2019) 076001. Realistic initial structures of different solid fractions have been constructed by the leveled-wave algorithm, approximating mixtures at the end of early-stage spinodal decomposition. The microstructural evolution during coarsening by surface diffusion was approximated by on-lattice kinetic Monte-Carlo simulation. The data sets refer to solid fractions from 0.22 to 0.50, providing for different initial connectivity of the bicontinuous structures. Coarsening at two temperatures, 900 K and 1800 K, explores two different degrees of surface energy anisotropy – more faceted at 900 K and more rough at 1800 K. Each structure takes the form of a face-centred cubic lattice with approximately 32 million sites. A site can be occupied by either void or atom. 3D periodic boundary conditions are satisfied. Tables list each structure's properties, and specifically the specific surface area, two different measures for the ligament size, the net topological genus as well as the scaled genus. The atom coordinate files may serve as the basis for geometry analysis and for atomistic as well as finite element simulation studies of nanoporous as well as spinodally decomposed materials. The data sets are accessible via the TORE repository at http://hdl.handle.net/11420/3253.

**Table:** Specifications table

Subject	Materials Science
Specific subject area	Nanoporous metals and atomistic simulation of microstructure evolution
Type of data	1, LAMMPS-style dump format, compressed in “zip” archive files. Those files contain atom position data of large nanoporous bodies with different solid fractions. The configurations represent either initial structures or structures in various stages of coarsening 2, tables listing parameters used for constructing the initial structures 3, tables listing geometric properties of initial and of coarsened structures
How data were acquired	On-lattice kinetic Monte Carlo (kMC) simulation, using the open source package SPPARKS
Data format	Raw and analyzed data
Parameters for data collection	The parameters for the numerical simulations (specifically, system sizes, number of iterations, solid fractions and temperatures) were selected as described and motivated in the original article.
Description of data collection	Initial configurations with solid fractions from 0.22 to 0.50 were generated numerically via the leveled-wave algorithm, were written to file and thereby collected. Parameters for the initial structures are provided in this article. Those structures served as the initial configurations for kMC runs with the open source kMC package SPPARKS. Coarsened NPG structures with simulations at 900 K and 1800 K were written to file and thereby collected. Specifically, these configurations included several intermediate stages of the coarsening as well as the final stage. Geometric properties, i.e. specific surface area, ligament size, genus and scaled genus for initial structures and coarsened structures are listed in the tables shown in this article.
Data source location	Hamburg University of Technology (TUHH), open source repository TUHH Open Research (TORE) at https://tore.tuhh.de/
Data accessibility	Repository name: TORE Data identification number: https://doi.org/10.15480/336.2393 Direct URL to data: http://hdl.handle.net/11420/3253
Related research article	Yong Li, Bao-Nam Dinh Ngô, Jürgen Markmann, Jörg Weissmüller Topology evolution during coarsening of nanoscale metal network structures Physical Review Materials 3 (2019) 076001. https://doi.org/10.1103/PhysRevMaterials.3.076001

**Table:** 

**Value of the Data** • The structures in this data set may be used as the starting configurations for investigations into the structure and properties of nanoporous gold, specifically where the interest is in how these properties vary when the solid fraction is varied during preparation, or when the mean structure size is varied during processing (for instance by annealing). • By their construction through the leveled-wave algorithm [[1], [2], [3]], the initial structures approximate not only dealloyed nanoporous gold but also the generic structure of mixtures at the end of early-stage spinodal decomposition [2]. All RVEs are therefore relevant as structure models for spinodally decomposed structures of different phase fraction and in various stages of coarsening. • The atom coordinates of the various nanoporous structures can be used directly as starting configurations in a variety of atomistic simulation schemes, for instance for studies of the mechanical properties by molecular dynamics or by finite-element modelling. Note that all data sets were consistently generated using periodic boundary conditions in all three dimensions of space, as is required for some of the above-mentioned simulation schemes.

## 1 Data

### 1.1 Nature of the data

The datasets in this work take the form of atom position coordinates in representative volume elements (RVEs) modelling bicontinuous structures that approximate experimental nanoporous gold made by dealloying. The data set comprises RVEs – designated as “initial” configurations – approximating the structure of dealloyed nanoporous gold in its as-prepared state, with various values of the solid (volume-) fraction, φ. These structures have been validated by comparison to experiment. Excellent agreement has been reported for the effective elastic behaviour (Young's modulus, Poisson's ratio) at different solid fractions [3]. The scaled density of topological genus has also been reported to agree with experiment [3]. The data set also comprises RVEs approximating the structure of nanoporous gold in various stages of coarsening. The time-evolution of the mean structure size and the evolution of the scaled topological genus with structure size have also been qualified as consistent with experiment [1].

**Fig. 1:**
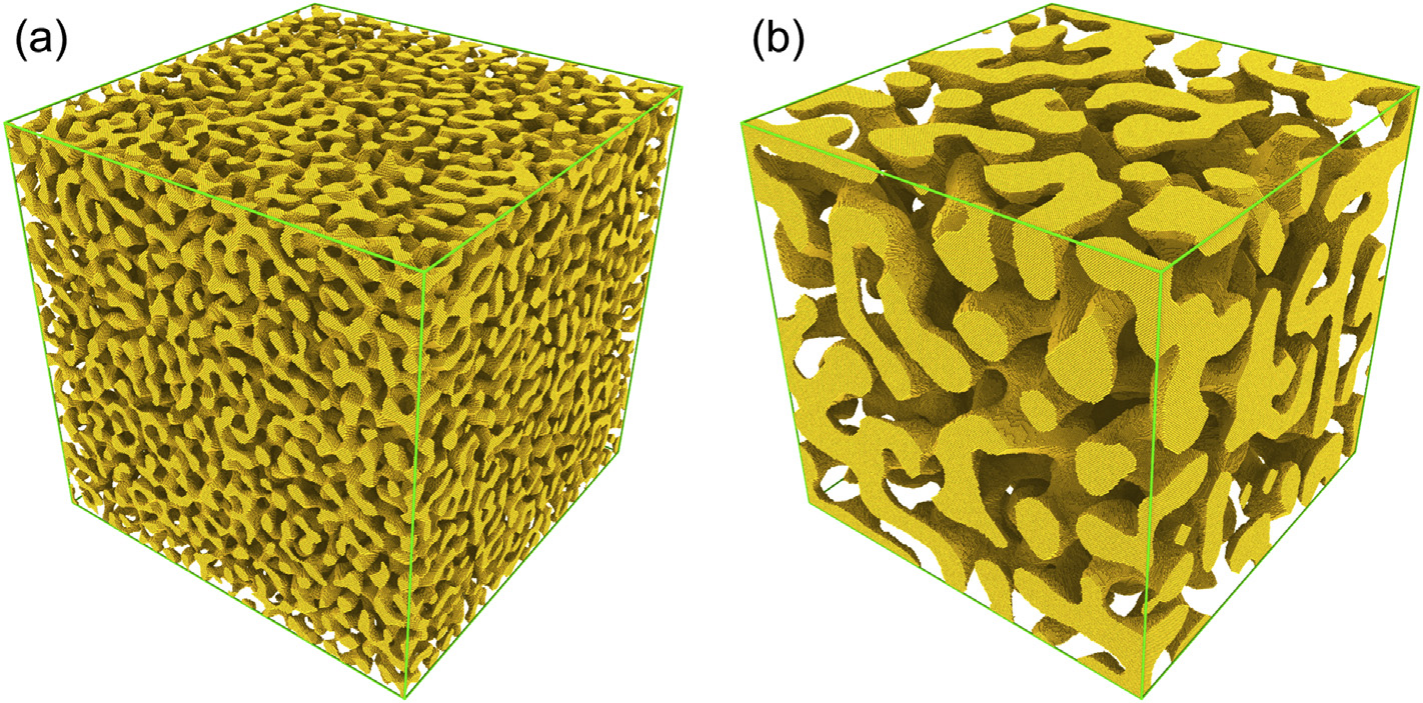
Microstructure of a typical RVE. Left (a), initial structure, generated by leveled wave algorithm. Right (b), final coarsened structure. The example shows the data from the files designated “200_30_449_120.txt.zip” and “200_30_449_120_1800.0532.txt.zip”, which refer to solid fraction 0.30 and coarsening temperature 1800 K.

Fig. 1 illustrates the microstructure in a typical RVE. Each RVE takes the form of a face-centred cubic (fcc) crystal lattice, with the lattice parameter of 408 pm and with 200 crystallographic unit cells along each edge, corresponding to 32 million sites in each RVE. The RVE's physical edge length is 81.6 nm.

The solid fractions of the initial configurations in the present data set are in the interval 0.22–0.50. Two measures for the characteristic microstructural length scale are reported, as explained in detail in Ref. [1]. The apparent ligament size, $L_{ap}$, is a measure for a mean diameter of the ligaments. The mean ligament spacing, $\tilde{L}$ , is a measure for the mean distance between neighbouring ligaments.

Table 1, Table 2 list two sets of different initial configurations, used for the coarsening runs (see below) at two different temperatures. Each batch collects a series of configurations with different φ. The first batch (Table 1) has identical apparent ligament diameter $L_{ap}$, independent of φ, but different mean ligament spacing $\tilde{L}$. The second batch (Table 2) has different $L_{ap}$ but identical $\tilde{L}$. Besides the measures for the characteristic structure size and the solid fraction, the tables also list the file name, the value of H and the surface area per solid volume, $S_{\text{V}}$. Furthermore, the table lists the net topological genus, G, of the RVE as well as the scaled genus, g. For definitions of these quantities, see Ref. [1].

**Table 1:** Initial configurations for the 900 K simulations: geometric and topological characteristics, as well as parameters used for construction. Solid fraction, φ; filename of the RVE; magnitude, H, of Miller-index square; wavelength, λ, underlying the Gaussian field; specific (per solid volume) area, $S_{V}$, of surface; apparent ligament size, $L_{ap}$; characteristic spacing, $\tilde{L}$, between ligaments; net topological genus, G, of the RVE; scaled genus, g.

φ	File name	H	λ	$S_{V}$	$L_{ap}$	$\tilde{L}$	G	g
no units			[nm]	[1/nm]	[nm]	[nm]	no units	*no units*
0.22	200_22_266_120.txt.zip	$\sqrt{266}$	5.00	1.69	2.36	5.66	1593	0.53
0.25	200_25_306_120.txt.zip	$\sqrt{306}$	4.66	1.72	2.33	5.23	2975	0.78
0.27	200_27_350_120.txt.zip	$\sqrt{350}$	4.36	1.79	2.24	4.86	4269	0.90
0.30	200_30_386_120.txt.zip	$\sqrt{386}$	4.15	1.79	2.23	4.60	5933	1.06
0.35	200_35_449_120.txt.zip	$\sqrt{449}$	3.85	1.77	2.25	4.24	9260	1.30
0.40	200_40_525_120.txt.zip	$\sqrt{525}$	3.56	1.77	2.26	3.89	13134	1.42
0.45	200_45_649_120.txt.zip	$\sqrt{649}$	3.20	1.82	2.20	3.44	19809	1.48
0.50	200_50_754_120.txt.zip	$\sqrt{754}$	2.97	1.80	2.22	3.16	25183	1.46

**Table 2:** Initial configurations for the 1800 K simulations. Analogous to Table 1.

φ	File name	H	λ	$S_{V}$	$L_{ap}$	$\tilde{L}$	G	g
no units			[nm]	[1/nm]	[nm]	[nm]	no units	*no units*
0.25	200_25_449_120.txt.zip	$\sqrt{449}$	3.85	2.13	1.87	4.24	5158	0.72
0.30	200_30_449_120.txt.zip	$\sqrt{449}$	3.85	1.95	2.05	4.24	7433	1.04
0.35	200_35_449_120.txt.zip	$\sqrt{449}$	3.85	1.77	2.25	4.24	9260	1.30
0.50	200_50_449_120.txt.zip	$\sqrt{449}$	3.85	1.34	2.98	4.23	11688	1.63

Each of the initial configurations listed in Table 1, Table 2 formed the basis for an atomistic kinetic Monte Carlo (KMC) simulation of coarsening by surface diffusion. Two series of runs explored coarsening at temperatures of 900 K and 1800 K, respectively. Snapshots of the atom coordinates where stored to disc at several points in time. The configuration files in the repository TORE contain this data. For the 900 K simulations, Table 4 identifies the corresponding filenames and it compiles information on the individual values of the coarsening time and of the geometric and topological characteristics. Table 5 provides the analogous information for the 1800 K simulations.

**Table 3:** Miller indices (h, k,l) for the individual values of H as listed in Table 1, Table 2.

H	h	k	l
266	11	9	8
	12	11	1
	13	9	4
	15	5	4
	16	3	1
306	11	11	8
	12	9	9
	13	11	4
	15	9	0
	16	5	5
	16	7	1
	17	4	1
350	13	10	9
	15	10	5
	15	11	2
	17	6	5
	18	5	1
386	12	11	11
	16	9	7
	16	11	3
	17	9	4
	19	4	3
	19	5	0
449	16	12	7
	17	12	4
	18	10	5
	18	11	2
	20	7	0
	21	2	2
525	16	13	10
	19	10	8
	20	10	5
	20	11	2
	22	5	4
649	18	15	10
	18	17	6
	18	18	1
	19	12	12
	21	12	8
	24	8	3
754	21	13	12
	23	12	9
	23	15	0
	24	13	3
	27	4	3
	27	5	0

**Table 4:** Contents of the individual configuration files for the 900 K simulations. Solid fraction, φ; filename of the snapshot; simulation time, t; surface area per solid volume, $S_{V}$; apparent ligament size, $L_{ap}$; characteristic spacing size, $\tilde{L}$; topological genus, G; and scaled genus, g of the snapshots microstructures.

φ	File name	t	$S_{V}$	$L_{ap}$	$\tilde{L}$	G	g
no units		[$v^{-1}$]	[1/nm]	[nm]	[nm]	no units	*no units*
0.22	200_22_266_120_0900.0504.txt.zip	2.0E+05	0.16	2.43	5.83	924	0.34
	200_22_266_120_0900.0602.txt.zip	2.0E+06	1.45	2.76	6.60	378	0.20
	200_22_266_120_0900.0606.txt.zip	4.0E+06	1.25	3.20	7.67	159	0.13
	200_22_266_120_0900.0618.txt.zip	1.0E+07	0.92	4.35	10.42	10	0.02
	200_22_266_120_0900.0704.txt.zip	2.2E+07	0.79	5.05	12.09	1	0.003
	200_22_266_120_0900.0716.txt.zip	6.8E+07	0.67	5.98	14.33	0	0
0.25	200_25_306_120_0900.0504.txt.zip	2.0E+05	0.17	2.40	5.39	2081	0.60
	200_25_306_120_0900.0602.txt.zip	2.0E+06	1.41	2.83	6.36	1127	0.53
	200_25_306_120_0900.0606.txt.zip	4.0E+06	1.14	3.51	7.87	503	0.45
	200_25_306_120_0900.0618.txt.zip	1.0E+07	0.84	4.74	10.64	82	0.18
	200_25_306_120_0900.0704.txt.zip	2.0E+07	0.73	5.46	12.25	30	0.10
	200_25_306_120_0900.0716.txt.zip	6.8E+07	0.62	6.46	14.50	10	0.06
0.27	200_27_350_120_0900.0504.txt.zip	2.0E+05	0.17	2.31	5.03	3054	0.71
	200_27_350_120_0900.0602.txt.zip	2.0E+06	1.39	2.89	6.27	1602	0.73
	200_27_350_120_0900.0606.txt.zip	4.0E+06	1.09	3.68	7.98	655	0.61
	200_27_350_120_0900.0618.txt.zip	1.0E+07	0.82	4.87	10.57	191	0.42
	200_27_350_120_0900.0704.txt.zip	2.0E+07	0.71	5.61	12.18	113	0.38
	200_27_350_120_0900.0716.txt.zip	6.8E+07	0.60	6.72	14.59	51	0.29
0.30	200_30_386_120_0900.0504.txt.zip	2.0E+05	0.17	2.31	4.76	4720	0.94
	200_30_386_120_0900.0602.txt.zip	2.0E+06	1.30	3.08	6.33	2330	1.09
	200_30_386_120_0900.0606.txt.zip	4.0E+06	0.99	4.06	8.36	955	1.03
	200_30_386_120_0900.0618.txt.zip	1.0E+07	0.75	5.31	10.92	354	0.85
	200_30_386_120_0900.0704.txt.zip	2.0E+07	0.68	5.92	12.18	230	0.77
	200_30_386_120_0900.0716.txt.zip	6.8E+07	0.59	6.79	13.98	158	0.79
0.35	200_35_449_120_0900.0504.txt.zip	2.0E+05	0.17	2.33	4.38	7936	1.23
	200_35_449_120_0900.0602.txt.zip	2.0E+06	1.17	3.41	6.41	3398	1.65
	200_35_449_120_0900.0606.txt.zip	4.0E+06	0.89	4.48	8.42	1417	1.56
	200_35_449_120_0900.0618.txt.zip	1.0E+07	0.72	5.57	10.48	741	1.57
	200_35_449_120_0900.0704.txt.zip	2.0E+07	0.64	6.28	11.82	509	1.55
	200_35_449_120_0900.0716.txt.zip	6.8E+07	0.54	7.45	14.01	319	1.62
0.40	200_40_525_120_0900.0504.txt.zip	2.0E+05	0.17	2.35	4.04	11707	1.42
	200_40_525_120_0900.0602.txt.zip	2.0E+06	1.06	3.78	6.49	4010	2.02
	200_40_525_120_0900.0606.txt.zip	4.0E+06	0.83	4.80	8.24	1919	1.98
	200_40_525_120_0900.0618.txt.zip	1.0E+07	0.67	6.01	10.32	992	2.01
	200_40_525_120_0900.0704.txt.zip	2.0E+07	0.58	6.85	11.76	667	2.00
	200_40_525_120_0900.0716.txt.zip	6.8E+07	0.50	7.98	13.70	450	2.13
0.45	200_45_649_120_0900.0504.txt.zip	2.0E+05	0.17	2.32	3.62	17728	1.55
	200_45_649_120_0900.0602.txt.zip	2.0E+06	0.94	4.24	6.63	3937	2.12
	200_45_649_120_0900.0606.txt.zip	4.0E+06	0.77	5.17	8.08	2224	2.16
	200_45_649_120_0900.0618.txt.zip	1.0E+07	0.61	6.53	10.21	1151	2.26
	200_45_649_120_0900.0704.txt.zip	2.0E+07	0.54	7.38	11.55	790	2.24
	200_45_649_120_0900.0716.txt.zip	6.8E+07	0.48	8.33	13.04	580	2.37
0.50	200_50_754_120_0900.0504.txt.zip	2.0E+05	0.17	2.40	3.40	22394	1.62
	200_50_754_120_0900.0602.txt.zip	2.0E+06	0.84	4.77	6.77	3682	2.11
	200_50_754_120_0900.0606.txt.zip	4.0E+06	0.72	5.57	7.91	2445	2.22
	200_50_754_120_0900.0618.txt.zip	1.0E+07	0.56	7.09	10.07	1228	2.30
	200_50_754_120_0900.0704.txt.zip	2.0E+07	0.49	8.10	11.50	821	2.30
	200_50_754_120_0900.0716.txt.zip	6.8E+07	0.43	9.28	13.18	603	2.54

**Table 5:** Contents of the individual configuration files for the 1800 K simulations. Analogous to Table 4.

φ	File name	t	$S_{V}$	$L_{ap}$	$\tilde{L}$	G	g
no units		[$v^{-1}$]	[1/nm]	[nm]	[nm]	no units	no units
0.25	200_25_449_120_1800.0320.txt.zip	1.0E+04	1.83	2.19	4.95	2091	0.47
	200_25_449_120_1800.0406.txt.zip	2.5E+04	1.44	2.78	6.29	773	0.35
	200_25_449_120_1800.0420.txt.zip	6.0E+04	1.10	3.64	8.24	175	0.18
	200_25_449_120_1800.0502.txt.zip	1.5E+05	0.89	4.51	10.20	52	0.10
	200_25_449_120_1800.0508.txt.zip	3.0E+05	0.76	5.25	11.88	19	0.06
	200_25_449_120_1800.0532.txt.zip	9.0E+05	0.63	6.38	14.43	2	0.01
0.30	200_30_449_120_1800.0320.txt.zip	1.0E+04	1.71	2.34	4.83	4515	0.94
	200_30_449_120_1800.0406.txt.zip	2.5E+04	1.33	3.01	6.21	2186	0.96
	200_30_449_120_1800.0420.txt.zip	6.0E+04	1.01	3.97	8.18	820	0.83
	200_30_449_120_1800.0502.txt.zip	1.5E+05	0.81	4.95	10.21	403	0.79
	200_30_449_120_1800.0508.txt.zip	3.0E+05	0.70	5.74	11.84	232	0.71
	200_30_449_120_1800.0532.txt.zip	9.0E+05	0.57	7.02	14.48	109	0.61
0.35	200_35_449_120_1800.0320.txt.zip	1.0E+04	1.59	2.52	4.74	6890	1.35
	200_35_449_120_1800.0406.txt.zip	2.5E+04	1.24	3.23	6.08	3670	1.52
	200_35_449_120_1800.0420.txt.zip	6.0E+04	0.93	4.31	8.11	1504	1.48
	200_35_449_120_1800.0502.txt.zip	1.5E+05	0.73	5.45	10.24	776	1.54
	200_35_449_120_1800.0508.txt.zip	3.0E+05	0.63	6.33	11.91	470	1.46
	200_35_449_120_1800.0532.txt.zip	9.0E+05	0.53	7.49	14.09	305	1.57
0.50	200_50_449_120_1800.0320.txt.zip	1.0E+04	1.23	3.25	4.62	10210	1.86
	200_50_449_120_1800.0406.txt.zip	2.5E+04	0.98	4.08	5.79	6059	2.17
	200_50_449_120_1800.0420.txt.zip	6.0E+04	0.72	5.56	7.90	2406	2.18
	200_50_449_120_1800.0502.txt.zip	1.5E+05	0.55	7.23	10.27	1134	2.26
	200_50_449_120_1800.0508.txt.zip	3.0E+05	0.48	8.25	11.71	773	2.29
	200_50_449_120_1800.0532.txt.zip	9.0E+05	0.43	9.30	13.21	573	2.43

For illustration, Fig. 1 shows the rendering of one exemplary structure in its initial state and at the end of coarsening simulation run.

It is noted that the configurations that are produced by coarsening may contain regions of solid that are disconnected from the percolating part of the solid. This is explained in detail in Ref. [1], see specifically figure 5b there. Some conceivable applications of the structures documented here may require that these disconnected regions be removed. As just one out of several options, the open source software OVITO [6] provides a simple and convenient way of achieving this.^1^

### 1.2 Format of the configuration data files

Each configuration file shows the distribution of two states, namely occupied or vacant, on the rigid periodic lattice. Each file represents a snapshot of the configuration at a particular moment in time, during the evolution of the microstructure. The files are formatted according to the “dump” file style of the molecular dynamics code LAMMPS [4,5]. The first 12 lines of each file are structured as in the following example:

ITEM: TIMESTEP
602 2.0e+06
ITEM: NUMBER OF ATOMS
32000000
ITEM: BOX BOUNDS
0 816
0 816
0 816
ITEM: ATOMS id type x y z
1 1 0 0 0
2 2 2.04 2.04 0
3 2 2.04 0 2.04
**… …**

Lines are separated by line feeds and separate entries on the same line are separated by blanks. The first line shows the header item “TIMESTEP”. The number “602” in line 2 is the identity number of the time step, in other words, of the snapshot at hand. The second number on line 2, namely “2.0e+06”, denotes the simulation time in units of $v^{-1}$. Here, ν is the attempt frequency [1]. The number “32000000” underneath the header item “NUMBER OF ATOMS” represents the total number of sites in the structure. Following the header item “BOX BOUNDS” are three lines, which contain two numbers each, here identically “0” and “816”. These describe the spatial coordinates at the two ends of each edge of the simulation box, in units of Ångstrom. Thereby, the three lines refer to the three orthogonal axes, which may be thought of as defining the x,y,z directions of the RVE. The last header item reads “ATOMS id type x y z”. This item defines the structure of the following lines, one line for each site of the crystal. This per-site information is structured as 5 columns. Here, “id” denotes the running number of the site, an integer. The “type” column contains the occupancy of the site, “1” for vacant or “2” for occupied. The entries “x”, “y”, “z” are the position coordinates of each site, again in units of Ångstrom. Initial configurations (listed in Table 1, Table 2) are stored with both vacant (type “1”) and occupied (type “2”) sites. To save repository resource, the coarsened structures (listed in Table 4, Table 5) are stored with occupied sites only.

## 2 Experimental design, materials, and methods

The methods used for generating the initial structures, for simulating the coarsening, and for analysing the data in terms of specific surface area and of topological genus are described in detail in Ref. [1].

The structures of the initial state – such as the example in Fig. 1a) – were generated by the leveled-wave algorithm as described in Refs. [1,3]. In brief, plane waves with identical wavelength λ but with differently oriented wave vectors $q_{i}$ and with random phase shifts were superimposed to generate the value of a Gaussian random field on each lattice site. That field was then binarized into “vacant” or “occupied” by taking a level cut at a threshold value selected for the desired phase fraction. Besides the binarization threshold of the level cut, the detailed geometry depends on the choice of wave vectors. We used $q_{i}\;=\;2\;\pi \lambda^{-1}\;\;\left\{h_{i},k_{i},l_{i}\right\}$, where the tuples $\left(h_{i},k_{i},l_{i}\right)$ consist of integers (the Miller indices) and are of fixed magnitude, H. In other words, $H=\sqrt{h_{i}^{2}+k_{i}^{2}+l_{i}^{2}}=const.$ For each choice of H, all $\left(h_{i},k_{i},l_{i}\right)$ consistent with that condition were used. Table 1, Table 2 list the H values for each data set, and Table 3 lists the corresponding sets of Miller indices.

Kinetic Monte Carlo (kMC) simulation explored the coarsening of the initial structures by surface diffusion. The kMC simulation used the open-source code SPPARKS [4,5], with input parameters as listed in detail in Ref. [1]. The code run in parallel on typically 64 cores, and the entire simulation used somewhat more than 10^6^ CPU hours.

The specific surface area, $S_{\text{V}}$ (area per volume of the solid phase), was computed by means of the open source software OVITO [6]. The apparent ligament size, $L_{ap}$, and characteristic spacing, $\tilde{L}$, were calculated by Eqs. (3) and (4) in Ref. [1]. The topological genus, G, which is equal to the Betti number $B_{1}$, was computed via open-source code CHomP [7] with periodic boundary conditions activated. For details see Ref. [1]. The scaled genus, g, was determined from Eq. (5) in Ref. [1].

## Footnotes

1 OVITO [6] provides the following sequence of commands: File → Load File → Add modification → Cluster analysis (Cutoff distance: 3; sort clusters by size) → Expression selection (operate on: particles; Boolean expression: Cluster ≥ 2) → DeleteSelected → File → Export File (File name: e.g. 200_22_266_120_0900.0716.txt.gz; Save as type: LAMMPS Dump File). Note that “zip” compressed files need to be uncompressed for loading into OVITO.
